# Effects of empagliflozin on right ventricular adaptation to pressure overload

**DOI:** 10.3389/fcvm.2023.1302265

**Published:** 2023-12-14

**Authors:** Julie S. Axelsen, Anders H. Nielsen-Kudsk, Janne Schwab, Steffen Ringgaard, Jens Erik Nielsen-Kudsk, Frances S. de Man, Asger Andersen, Stine Andersen

**Affiliations:** ^1^ Department of Cardiology, Aarhus University Hospital and Department of Clinical Medicine, Aarhus University, Aarhus, Denmark; ^2^ MR Research Centre, Aarhus University, Aarhus, Denmark; ^3^ PHEniX Laboratory, Department of Pulmonary Medicine, Amsterdam UMC, Locatie VUmc, Amsterdam, Netherlands

**Keywords:** right ventricular failure, pulmonary hypertension, sodium-glucose co-transport-2 inhibitors, empagliflozin, pulmonary trunk banding, rat model

## Abstract

**Background:**

Right ventricular (RV) failure is the prime cause of death in patients with pulmonary arterial hypertension. Novel treatment strategies that protect the RV are needed. Empagliflozin, a sodium-glucose co-transporter-2 inhibitor, shows cardioprotective effects on the left ventricle in clinical and preclinical studies, but its direct effects on RV remain elusive. We investigated the effects of empagliflozin on RV dysfunction induced by pulmonary trunk banding (PTB).

**Methods:**

Male Wistar rats (116 ± 10 g) were randomized to PTB or sham surgery. One week after surgery, PTB animals received empagliflozin mixed into the chow (300 mg empagliflozin/kg chow; PTB-empa, *n* = 10) or standard chow (PTB-control, *n* = 10). Sham rats (Sham, *n* = 6) received standard chow. After five weeks, RV function was evaluated by echocardiography, cardiac MRI, and invasive pressure-volume measurements.

**Results:**

PTB caused RV failure evident by decreased cardiac output compared with sham. PTB-empa rats had a 49% increase in water intake compared with PTB-control yet no differences in hematocrit or blood glucose. Treatment with empagliflozin decreased RV end-systolic pressures without any changes in RV cardiac output or ventricular-arterial coupling (Ees/Ea). The decrease in RV end-systolic pressure was complemented by a slight reduction in RV cross sectional area as a sign of reduced hypertrophy. Load-independent measures of RV systolic and diastolic function were not affected in PTB-empa rats compared with PTB-control.

**Conclusion:**

Empagliflozin treatment reduced RV end-systolic pressure in RV failure induced by pressure overload. Further studies are needed to elucidate whether this simply relates to a diuretic effect and/or additional independent beneficial RV effects.

## Graphical Abstract

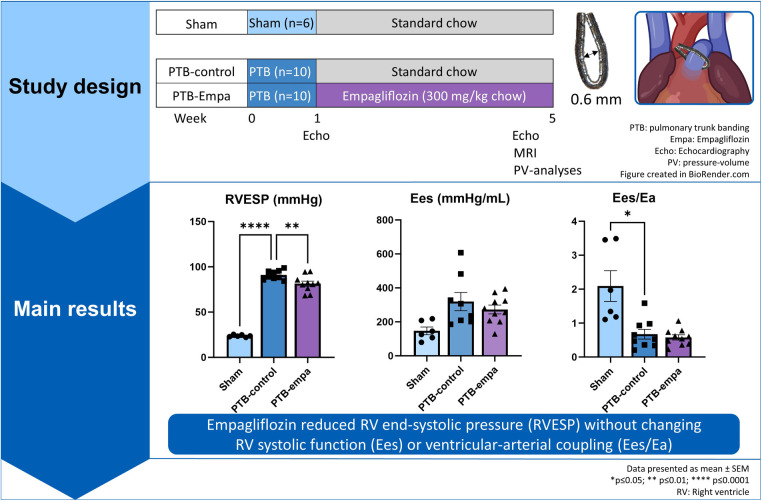

## 1. Introduction

Right ventricular (RV) failure is the predominant cause of death in patients with pulmonary arterial hypertension (PAH) (1–3). Current therapeutic agents targeting PAH focus on lowering RV afterload by dilating the pulmonary vessels, but they do not directly affect RV dysfunction. We do however know, that RV function can continue to decline even after a reduction in pulmonary vascular resistance (4). Therefore, it is essential to search for new therapeutic drugs that will have direct protective effects on RV function.

The new anti-diabetic group of drugs, sodium-glucose co-transporter-2 (SGLT2) inhibitors, have shown to reduce the risk of hospitalization for heart failure (HF) independent of atherosclerotic disease (5–7). They are now recommended by the European Society of Cardiology for patients with HF with reduced ejection fraction (8) and as from 2023 also for patients with HF with preserved ejection fraction (9). Despite massive research, the underlying mechanisms of SGLT2 inhibitors’ cardioprotective effects remain elusive (10). Beside their glycosuric and natriuretic effects, several mechanisms have been proposed relating to the left ventricle (LV) e.g., optimization of cardiac energy metabolism (11–15), reduction of cardiac hypertrophy and oxidative stress (16), as well as increased capillarization (17). Regarding diastolic function, SGLT2 inhibitors reduce cardiac and extracardiac fibrosis (11, 18–20), and empagliflozin reduced diastolic tension in myocardial fibers from end-stage HF patients and rats by increasing phosphorylation of the myofilament protein titin (21, 22).

Fundamental differences between LV and RV hamper direct translation. The promising results on the LV paved the way for investigating SGLT2 inhibitor's effects on RV failure development. So far, the RV effects have been ambiguous. The two SGLT2 inhibitors, empagliflozin and dapagliflozin, showed promising effects in the monocrotaline (MCT) model of experimental PH by reducing RV hypertrophy, RV fibrosis, pulmonary artery and RV pressures, as well as improving calcium-handling (19, 23, 24). In the pulmonary trunk banding (PTB) model, an isolated model of RV failure, dapagliflozin reduced RV mass, improved nutrient sensing, and increased contractility (25). On the other hand, Li et al. (26) did not find any effects on RV function or disease development after dapagliflozin treatment in the PTB or MCT models.

Though the different SGLT2 inhibitors have similar mechanisms of action they show differences in pharmacodynamic and pharmacokinetic properties (10). To date the direct cardiac effects of empagliflozin on RV function remain unknown. We investigated the effects of empagliflozin on RV systolic and diastolic adaptation to pressure overload induced by PTB.

## 2. Methods

### 2.1. Animals

The rats were treated according to Danish national guidelines, and all experiments were approved by the Institutional Ethics Review Board, the Danish Animal Experiments Inspectorate, and conducted in accordance with the Danish law for animal research (authorization number 2021-15-0201-00928, Ministry of Environment and Food of Denmark). Male Wistar rats (Janvier Labs, Hannover) were given *ad libitum* access to drinking water and standard rat chow (Altromin #1324; Altromin, Lage, Germany) with or without empagliflozin. The rats were housed two per cage (open-top, type III cage) with Abedd Bedding material and environmental enrichment including shelter, gnawing brick, and nesting material. The cages were kept in a cabinet (Scantainer) in a room with a 12-hour light-dark cycle and 21˚C. The veterinary staff checked the health status daily by assessing activity, respiration, and fur condition.

### 2.2. Study design

**Figure 1:**
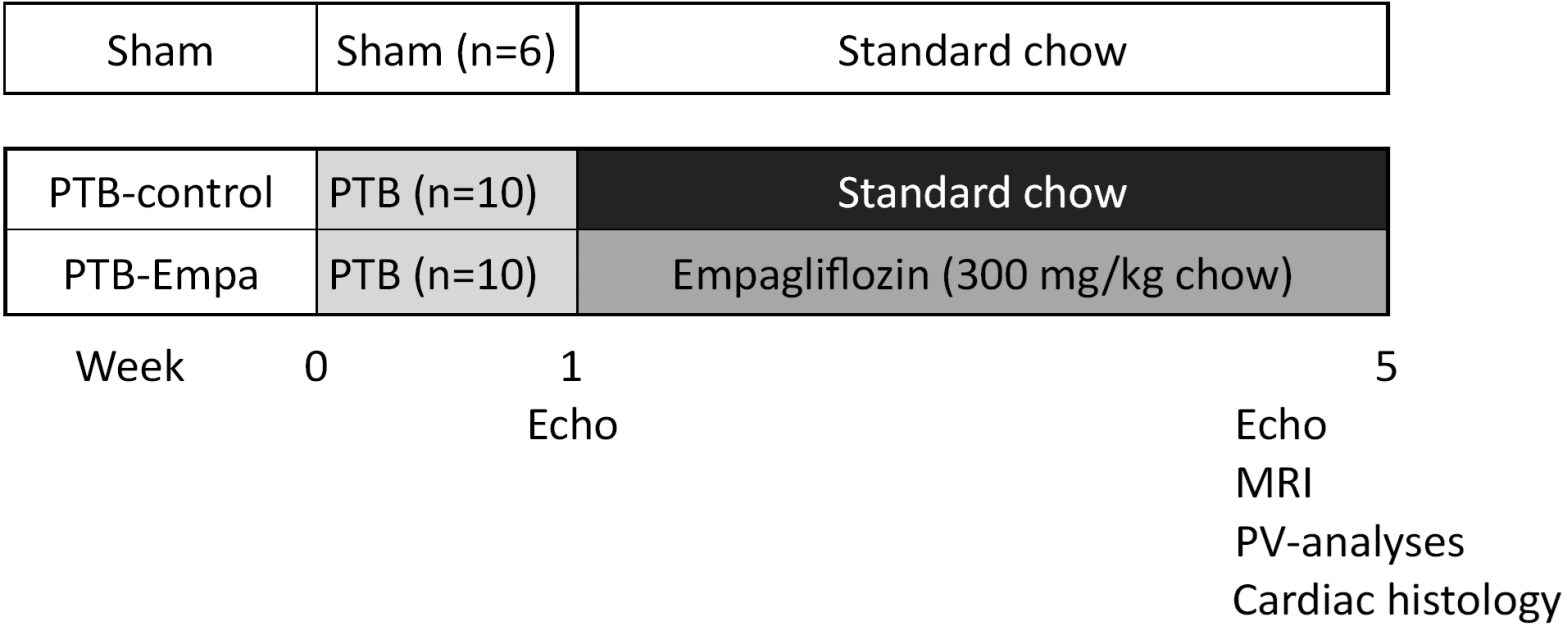
Study design. Male Wistar rat weanlings were randomized to sham or pulmonary trunk banding (PTB) surgery. One week after the surgery, a baseline echocardiography was performed, and treatment was initiated. Five weeks after the PTB surgery, cardiac function was evaluated by clinically relevant methods including echocardiography, MRI, and invasive pressure-volume measurements. The rats were euthanized, and histochemical analyses were performed on the heart.

Male Wistar rat weanlings (116 ± 10 g) were randomized to either pulmonary trunk banding (PTB) or sham surgery after one week of acclimatization. Treatment, standard chow (PTB-control, *n* = 10) or empagliflozin mixed into standard chow (PTB-empa, *n* = 10), was initiated one week after the surgery and after a baseline echocardiography had been performed. The empagliflozin concentration was 300 mg empagliflozin/kg chow (by Brogaarden, Lynge, Denmark) aiming for a dosage of 20 mg/kg/day based on pilot studies and previous reports (27, 28). Total treatment duration was 4 weeks. Sham rats (Sham, *n* = 6) received standard chow. The rats’ weight and their consumption of chow and water were measured twice weekly. Five weeks after the PTB surgery, RV systolic and diastolic function were evaluated by clinically relevant methods including echocardiography, MRI, and invasive pressure-volume measurements. Afterwards, the hearts of the rats were excised, and histochemical analyses were performed to assess RV remodeling (Figure 1). On each day of experiments, the order of rats was randomized.

### 2.3. Pulmonary trunk banding (PTB)

The PTB surgery was performed as described previously (29). Briefly, the rats were anesthetized (sevoflurane, Sevorane, AbbVie Inc, 7% induction, 3.5% maintenance in a 2:1 O_2_/air mix), intubated, and connected to a rodent ventilator (Ugo Basile 7025, rodent ventilator, respiration frequency of 76 per minute and tidal volume adjusted by weight). The pulmonary trunk was carefully isolated through a lateral thoracotomy, and a modified ligating clip applier was used to compress the titanium clip to a preset inner diameter of 0.6 mm around the pulmonary trunk. For analgesia during surgery, the rats were injected with s.c. 0.1 mg/kg buprenorphine (Temgesic, Indivior Europe Limited, Ireland) and carprofen 5 mg/kg (ScanVet Animal Health, Fredensborg, Denmark). The following three days, the rats were treated with buprenorphine in the drinking water (7.4 µg/ml). The sham rats underwent the same surgery except for the clip application.

### 2.4. Evaluation of hemodynamic and anatomic measures

#### 2.4.1. Echocardiography

Transthoracic echocardiography was performed one and five weeks after surgery with a Vevo 2100 Imaging System (VisualSonics Inc, Toronto, ON, Canada) scanning at 14–21 MHz using a MS250 line array transducer. Following views were obtained: Parasternal long axis view to assess inner diameter of the pulmonary trunk (PT), velocity time integral (VTI), and calculate stroke volume (SV) (SV = (PT diameter/2)_2_·π·VTI); Apical 4 chamber views with tricuspid annular plane systolic excursion (TAPSE), pulse wave Doppler at the tip of the tricuspid leaflet, and tissue Doppler imaging of the lateral tricuspid annulus. All parameters were measured in three consecutive heart cycles to minimize beat-to-beat variation and analyzed off-line (Vevo® 2100 software v. 5.6.0, Fujifilm Visualsonics Inc, The Netherlands).

#### 2.4.2. Cardiac MRI

A 9.4 Tesla Agilent MR system with a volume transmit/receive rat coil was used to measure RV and right atrium (RA) volumes by manually drawing the endocardium using Segment v3.3 R9405e (http://segment.heiberg.se) (30) on a set of short axis cine images (slice thickness 1.5 mm and in-plane resolution 0.2 mm). Cardiac output was obtained with a phase contrast flow measurement in the pulmonary artery (slice thickness 1.5 mm and in-plane resolution 0.23 mm). Flow was calculated with the specially made analysis software Siswin.

#### 2.4.3. Invasive pressure-volume measurements

To assess blood glucose, sodium, and hematocrit, a small blood sample was taken from the tale shortly after the non-fasting rats had been anesthetized and intubated. Afterwards, the rats were injected with 50 units of heparin i.m. (Heparin, Leo Pharma A/S, Denmark) to prevent blood clotting during the procedure. After stabilization, systemic blood pressures were measured in the right carotid artery. RV pressure-volume loops were obtained by a conductance catheter (SPR-869, Millar Instruments, Texas) installed in the RV through the apex using an open-chest approach. Briefly, the diaphragm was accessed from the abdomen and the ribs cut in two straight lines from processus xiphoideus to the axils. After carefully angling the chest plate, the pericardium was removed, and the catheter inserted into the RV through a small needle puncture at the apex. The signals were processed in Powerlab 16/35 (AD Instruments, UK) with a sampling rate of 2 kHz. RV steady state pressures were obtained followed by pressure volume loops at declining preloads by gradually occluding the inferior vena cava. The conductance signal was calibrated using MRI volume measures, and Labchart (AD Instruments, UK) were used to calculate both load-independent and load-dependent measures of RV contractility and diastolic function.

#### 2.4.4. Euthanasia

The heart was quickly excised and cut. The atria and ventricles were weighed individually. RV/(LV + septum (S)) was used as measure of RV hypertrophy. Thoracic and abdominal cavities were assessed for pleural effusion and ascites (cut off >1 g of fluid), and the liver was weighed and examined for dark discoloration (nutmeg liver). Remaining organs were weighed.

All images and analyses were performed with the observer blinded to the source of the sample.

### 2.5. Analyses of cardiac tissue

Cardiac tissue was immersion fixated in 10% formalin and embedded in paraffin within 72 h. Sections of 3 µm were stained with hematoxylin & eosin or collagen specific picrosirius red. Whole slide scanning was performed on NanoZoomer 2.0HT (Hamamatsu Photonics K.K). The RA and RV cardiomyocyte cross sectional area (CSA) were analyzed using Nanozoomer Digital Pathology software (NDP.view 2.9.29, Hamamatsu Photonics K.K) and expressed as the average CSA of minimum 23 RA or 35 RV cardiomyocytes and secondarily pooled within the groups to investigate cell size heterogeneity. The measured cardiomyocytes were distributed in the whole section and only selected when cut transversally at the level of the nuclei. RV fibrosis was expressed as the mean percentage tissue area positive for collagen measured over a minimum of six randomly chosen areas by adjusting color thresholds in ImageJ (Rasband, W.S., ImageJ, U. S. National Institutes of Health, Bethesda, Maryland, USA, https://imagej.nih.gov/ij/, 1997–2018). Areas with contaminations, artefacts, or larger vessels were excluded. All histological analyses were performed by an operator blinded to the source of the sample.

### 2.6. Statistics

All statistical analyses were performed using GraphPad Prism 9.4.0 for Windows (GraphPad Software, San Diego, California USA, www.graphpad.com) with *p* < 0.05 considered statistically significant. Data were tested for normal distribution by the Shapiro Wilk test and non-parametric tests were used if non-normally distributed. Quantitative data are expressed as median [interquartile range] in tables and mean ± standard error of mean (SEM) in scatterplots. Analyses were performed using one-way ANOVA with Bonferroni post-hoc comparison or a non-parametric Kruskal Wallis test to evaluate the model (sham vs. PTB-control) and the effects after empagliflozin treatment (PTB-control vs. PTB-empa). The Brown-Forsythe ANOVA test was used when there were significant differences in standard deviation (SD). Dichotomous outcomes were compared between groups by Fisher's exact test. 2-way ANOVA was used to show interaction of empagliflozin over time.

A power calculation was performed to determine group size. Cardiac index (CI, cardiac output (CO)/body surface area (BSA)) measured by echocardiography was chosen as primary outcome. Pilot studies showed a standard deviation within the PTB-group of 0.1694 ml/s/m^2^. Based on previous literature, we expected a 7.5% increase in CI. With a significance level of 0.05 and a power of 0.8, nine rats were required in each PTB group. Due to a mortality risk of approximately 10%, we decided to include ten rats in each PTB group.

## 3. Results

With a mortality rate of 16% during PTB surgery, a total of 31 rats underwent surgery and 26 rats were included. One PTB-control rat died during MRI due to extensive hydrothorax caused by RV failure. There were no significant differences in mortality between the groups.

### 3.1. Effects of pulmonary trunk banding (PTB) on RV function

**Figure 2:**
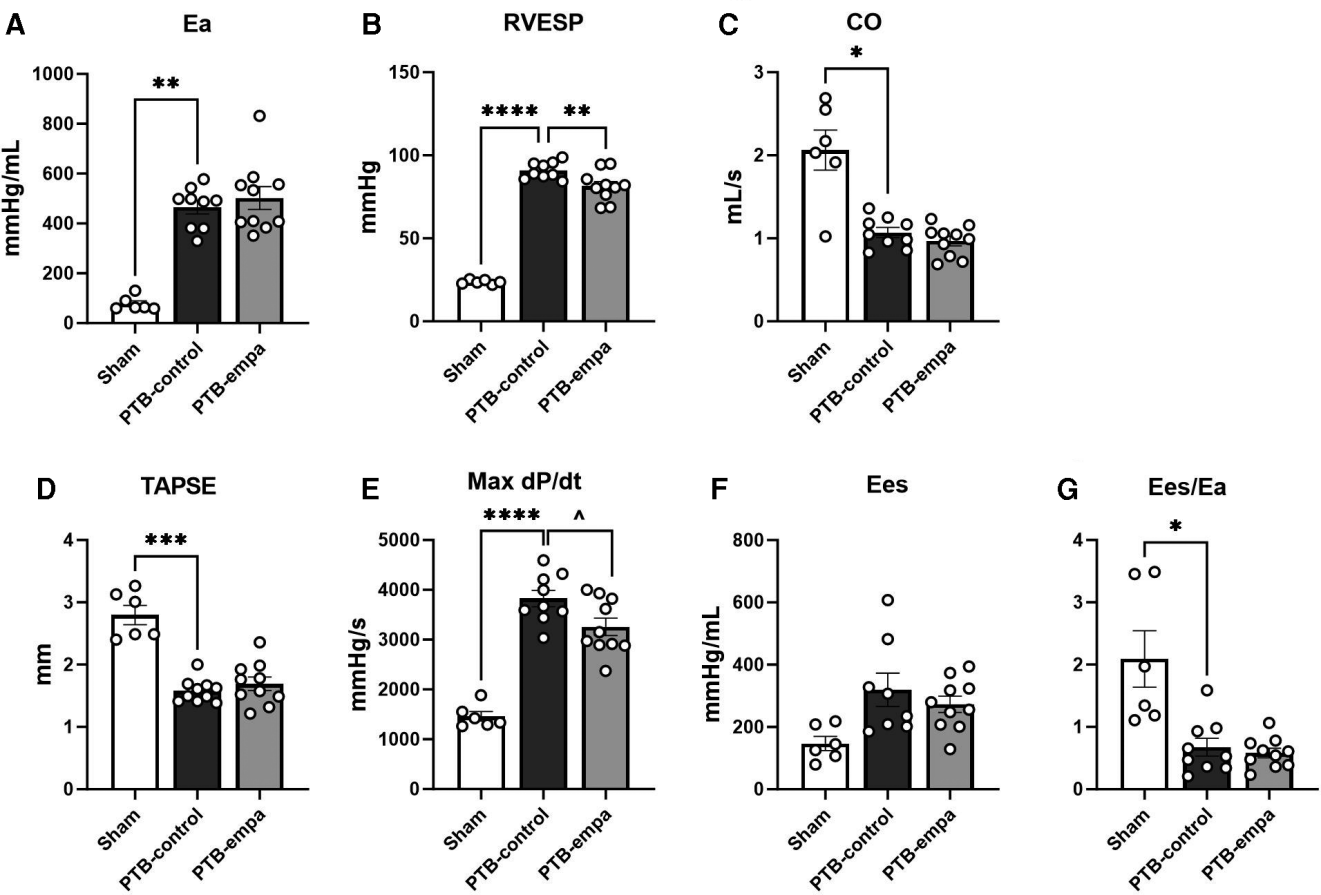
Pulmonary trunk banding and empagliflozin's effect on right ventricular systolic dysfunction at end-of-study. PTB: pulmonary trunk banding, empa: empagliflozin. (**A**) Arterial elastance. (**B**) Right ventricular (RV) end-systolic pressure. (**C**) Cardiac output. (**D**) Tricuspid annular plane systolic excursion (TAPSE). (**E**) First derivative (maximal) of right ventricular systolic pressure. One PTB-control excluded. (**F**) End-systolic elastance. (**G**) Ventricular-arterial coupling. Results are expressed as scatterplots with mean ± SEM. *p < 0.05; **p < 0.01; ***p < 0.001; ****p < 0.0001 PTB-control vs. sham. ^*p* < 0.05 PTB-empa vs. PTB-control.

PTB increased RV afterload (arterial elastance, Ea) six-fold and RV end-systolic pressures (RVESP) four-fold in PTB-control rats compared with sham. RV dysfunction was evident by decreased RV cardiac output (CO), RV ejection fraction (EF), and tricuspid annular plane systolic excursion (TAPSE). DP/dt_max_, a load-dependent measure of contractility, was increased in PTB-control rats compared with sham. The load-independent measure of contractility, RV end-systolic elastance (Ees), showed similar trend though non-significantly. Still, the increase in contractility was not sufficient to maintain the ventricular-arterial coupling (Ees/Ea) (Table 1, Figure 2).

**Table 1:** Anatomic and hemodynamic data at end-of-study.

	Sham *n* = 6	PTB-control *n* = 10	PTB-empa *n* = 10
Anatomical data			
BW at end-of-study (g)	328 [324–339]	336 [294–325]	292 [282–333]
RV weight (mg)	210 [198–218]	430 [370–488]****	420 [380–485]
LV + S weight (mg)	705 [673–748]	825 [740–885]**	765 [708–815]
RV fibrosis (%)	4.2 [3.9–4.7]	6.2 [5.3–6.8]**	6.3 [6.0–6.6]
EC manifestations	0 (0)	2 (20)	2 (20)
TR	0 (0)	5 (50)	6 (60)
Empagliflozin effects			
Chow consumption/day (g)	23.3 [23.1–23.8]	22.6 [21.7–24.2]	23.3 [22.1–25.7]
Water consumption/day (g)	30.5 [29.9–31.6]	27.8 [26.1–28.5]**	49.9 [38.0–43.1]^∧∧∧∧^
Blood glucose (mmol/L)	10.1 [9.8–11.0]	10.0 [9.5–10.8]	9.5 [9.1–11.1]
Blood sodium (mmol/L)	139.5 [138.5–140.3]	140.0 [138.0–140.5]	138.0 [137.0–138.3]
Hematocrit (%)	47 [45–49]	47 [46–49]	49 [47–51]
Hemodynamic measures			
RV systolic pressure (mmHg)	25 [24–27]	88 [86–96]****	80 [71–97]
RV EF (%)	73 [69–75]	42 [39–49]****	48 [33–52]
Tricuspid S’ (mm/s)	66 [62–68]	43 [40–48]****	39 [35–43]
HR (bpm)	453 [398–479]	359 [338–380]***	373 [314–387]
RV SV (ml)	0.37 [0.24–0.40]	0.19 [0.17–0.23]***	0.17 [0.14–0.20]
MAP (mmHg)	128 [81–138]	121 [118–131]	113 [103–123]

PTB, pulmonary trunk banding; empa, empagliflozin; BW, body weight; RV, right ventricle; LV + S, left ventricle + septum; RV, right ventricle; EC, extracardiac; EC manifestations, nutmeg liver, ascites and/or hydrothorax; TR, tricuspid regurgitation; EF, ejection fraction; HR, heart rate; SV, stroke volume; MAP, mean arterial pressure. Data are presented as median [interquartile range] or *n* (%).

** *p* < 0.01.

*** *p* < 0.001.

**** *p* < 0.0001 PTB-control vs. sham.

^∧∧∧∧^ *p* < 0.0001 PTB-empa vs. PTB-control.

**Figure 3:**
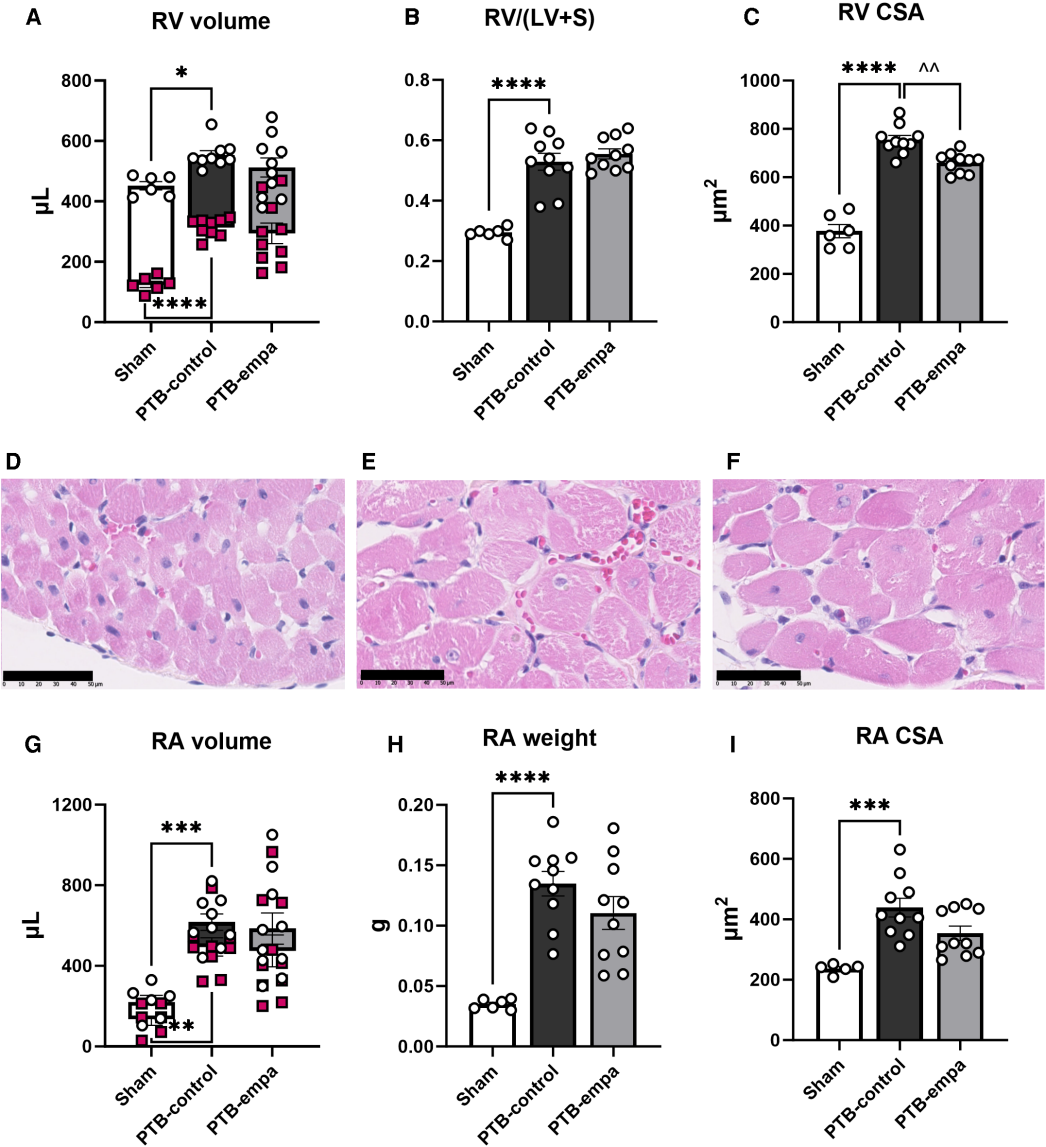
Effects of pulmonary trunk banding and empagliflozin on RV and RA dilatation and hypertrophy. PTB: pulmonary trunk banding, empa: empagliflozin. (**A**) RV end-systolic (red markings) and end-diastolic volume (white markings). (**B**) Fulton index (right ventricle (RV)/left ventricle (LV) and septum (**S**). (**C**) RV cross sectional area (CSA). Histological RV sections stained with hematoxylin & eosin from (**D**) Sham, (**E**) PTB-control, and (**F**) PTB-empa, black scale bar equals 50 µm. (**G**) RA end-systolic (red markings) and end-diastolic volume (white markings). (**H**) Right atrial (RA) weight. (**I**) RA cross sectional area (CSA), data from one sham animal is missing. Results are expressed as scatterplots with mean ± SEM. **p < 0.01; ***p < 0.001 PTB-control vs. sham. ^^*p* < 0.01 PTB-empa vs. PTB-control.

The increased RV pressures in PTB-control rats caused RV dilatation with increased RV volumes and RV hypertrophy evident by increased RV/(LV + S) weight ratio and RV cross sectional area (CSA) compared with sham rats. The RA was similarly affected with increased RA volumes, weight, and CSA, indicating a backward build-up of pressure (Figure 3).

**Figure 4:**
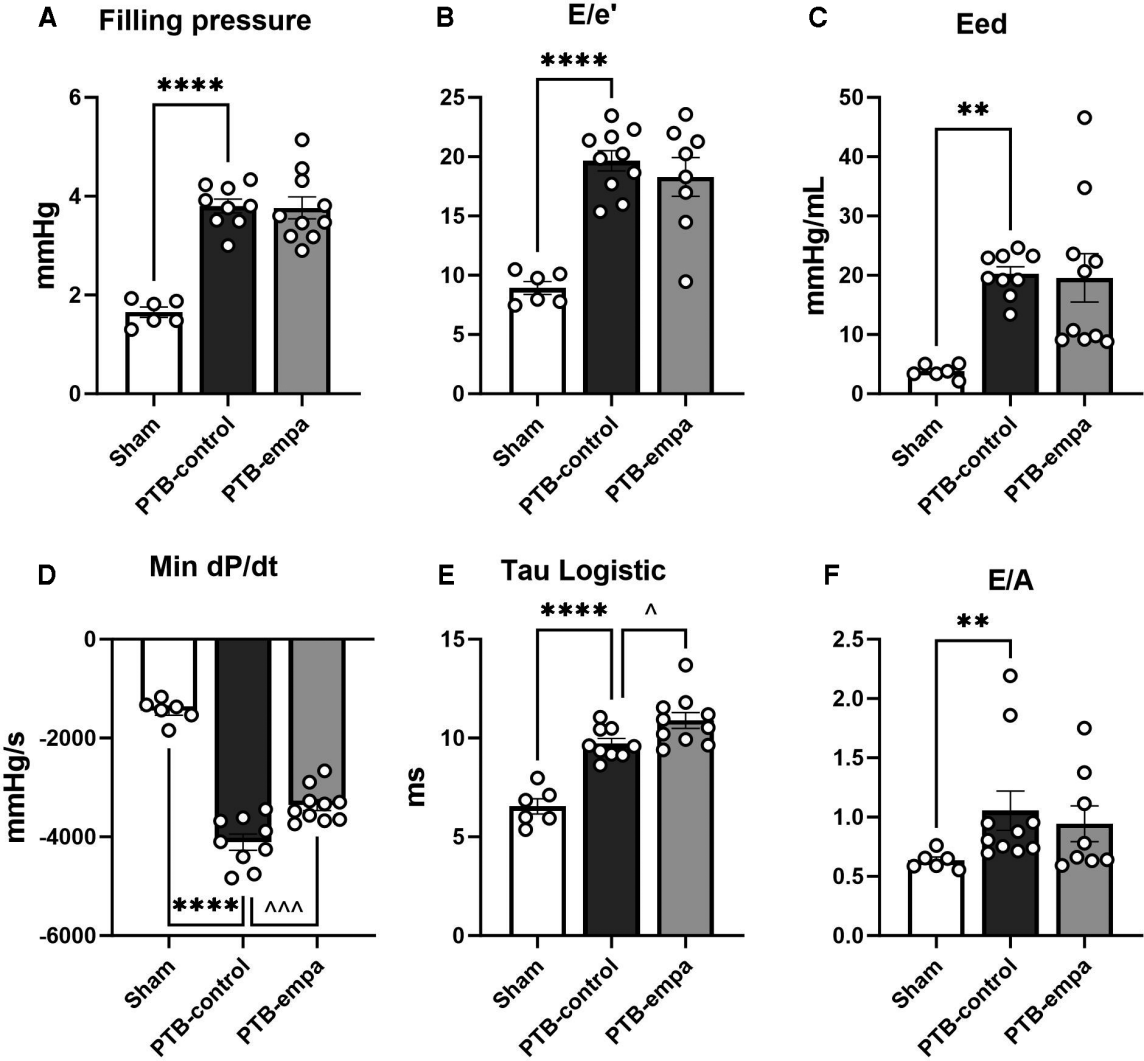
Effects of pulmonary trunk banding and empagliflozin on RV diastolic dysfunction at end-of-study. PTB: pulmonary trunk banding, empa: empagliflozin. (**A**) Right ventricular (RV) filling pressure as (end-diastolic pressure)–(beginning of diastolic pressure). (**B**) RV filling pressure as tricuspid valve E/e’. Two PTB-empa are missing due to EA fusion. (**C**) RV end-diastolic elastance. (**D**) First derivative (minimal) of RV systolic pressure. (**E**) The relaxation time constant, Tau logistic. (**F**) Ratio of trans tricuspid valve flow velocities. Two PTB-empa rats missing due to EA fusion. Results are expressed as scatterplots with mean ± SEM. **p < 0.01; ****p < 0.0001 PTB-control vs. sham. ^*p* < 0.05; ^^^*p* < 0.001 PTB-empa vs. PTB-control.

Diastolic dysfunction was evident in PTB-control rats compared with sham as both invasive and non-invasive (E/e’) filling pressures were increased. Stiffening of the RV was further evident by increased load-independent end-diastolic elastance (Eed), Tau logistic, and E/A. PTB further increased load-dependent dP/dt_min_ (Figure 4).

### 3.2. Effects of empagliflozin on RV function

At baseline, one week after surgery, no weight differences between the two PTB groups were observed (Supplementary Table S1). At end-of-study, PTB-empa rats weighed 8% less than PTB-control, though only a significant difference when observed over time (Supplementary Figure S1). Based on chow consumption in each cage adjusted by the weight of the rats, the median calculated empagliflozin dosage was 30.2 mg/kg/day [28.9–31.4]. Empagliflozin treatment increased water consumption by 49% compared with PTB-control, which is a well-known effect of empagliflozin treatment. Other well-known effects of empagliflozin treatment are glucosuria causing diuresis and natriuresis. In present study however, PTB-empa rats showed stable hematocrit, blood glucose, and sodium compared with PTB-control rats (Table 1).

On echocardiographic parameters at baseline, no differences between the two PTB groups were observed (Supplementary Table S1). At end-of-study, the PTB-empa rats had similar afterload, but RVESP was 11% lower than in PTB-control rats corresponding to only a 10 mmHg pressure reduction. A similar trend was observed in maximum RVSP. No differences in cardiac output were observed at end-of-study. The load-dependent dP/dt_max_, a measure of contractility, was less affected in PTB-empa with similar trend in Ees without worsening the ventricular-arterial coupling (Ees/Ea) (Figure 2).

The reduced RVESP did not cause changes in RV/(LV + S) weight ratio, but a slight decrease in RV CSA was observed (Figure 3). This was further supported when the RV CSA data were pooled (Supplementary Figure S2). Empagliflozin treated rats exhibited large variations in RV end-diastolic and end-systolic volumes. Same trends were seen in RA weight, RA CSA, and RA volumes. Regarding diastolic parameters, no differences in RV fibrosis or filling pressures were observed in PTB-empa rats compared with PTB-control. Though, a less affected dP/dt_min_ was observed in the PTB-empa rats (Table 1, Figures 3, 4).

## 4. Discussion

We investigated the cardiac effects of empagliflozin on RV adaptation to pressure overload and showed that:

- Treatment with empagliflozin increased water intake but did not reduce hematocrit or blood glucose.
- Empagliflozin treatment caused a mild reduction in RV end-systolic pressure.
- Empagliflozin treatment did not alter systolic function (Ees) or ventricular-arterial coupling.
- Empagliflozin had no toxic or adverse effects on the failing RV.

### 4.1. PTB caused RV failure and adverse remodeling

PTB surgery caused increased afterload, to which the RV adapted by increasing load-dependent contractility dP/dt_max_, RV volumes, and hypertrophy measured as RV/(LV + S) weight ratio and RV CSA. This correlates well with clinical RV failure development seen in patients with PAH, where RV failure is associated with disturbed ventricular-arterial coupling, diastolic deterioration, and increased contractility (31). This finding is confirming the PTB model's applicability to investigate disease mechanisms underlying RV failure and to identify potential new therapeutic drugs.

### 4.2. Systemic effects of empagliflozin treatment

PTB-empa rats showed obvious signs of the empagliflozin treatment by increased water intake caused by glucosuria. In present study, the PTB-empa rats were able to sustain their hematocrit and blood glucose levels with a reduced body weight compared with PTB-control rats. The PTB-empa rats did not show signs of kidney dysfunction (Supplementary Table S2). Others have described similar effects in rodents including the diuretic effect, stable hematocrit, and weight reduction after treatment with an SGLT2-inhibitor (25, 32).

### 4.3. The effects of empagliflozin treatment on RV function

Some of the clinical characteristics of RV failure are fluid retention evident by peripheral oedema, ascites, and effusion. This is typically managed by diuretics and by minimizing fluid and sodium intake (31, 33). However, studies of the diuretics’ effects in patients with RV failure are lacking, the general understanding and clinical experience coincide, that diuretics reduce RV preload and thereby improving cardiac output and RV function (34). In present study, the diuretic effects of empagliflozin may have caused the small reduction seen in RVESP which secondarily caused less affected load-dependent measures of contractility (dP/dt_max_) and relaxation (dP/dt_min_). This could also be signs of a potential negative effect of empagliflozin. However, the fact that we did not see any worsening of the ventricular-arterial coupling in the PTB-empa rats compared with PTB-control supports that empagliflozin did not worsen RV function, but simply imply that the RV did not need further enhancement. The same applies for the small increase seen in Tau logistic in the empagliflozin treated rats which imply a slower active relaxation rate and thereby potential worsening of RV diastolic function. However, as none of the other diastolic parameters worsened it may be difficult to interpret a single diastolic measure, as these previously have shown to be incongruent with each other over time (35). More knowledge is needed to elucidate whether this single increase in Tau logistic depicts a potential worsening of diastolic function at this disease stage. Accordingly, the diuretic effect may explain some of the RV effects seen in present study, but we cannot exclude other mechanisms might play a role, as recent studies describe a direct cardiac effect of empagliflozin on cardio myocytes and SGLT2-knock out mice without affecting systemic SGLT2 function (36–38). Either way, we solely see a slight disease reduction and not a normalization of RV function in present study, emphasizing the need for further investigation of empagliflozin's role as a potentially new therapy complimenting current RV failure management.

Chowdhury et al. (23) found similar effects in MCT rats treated with empagliflozin with reduced RVSP and RV hypertrophy together with reduced mean pulmonary artery pressure and prevented adverse pulmonary remodeling. They too showed reduced RV collagen content, while we in present study did not see changes in RV fibrosis. In the MCT model it is not possible to distinguish the direct cardiac effects from effects secondary to afterload reduction, which may explain some of the differences. In present study, we did not see significant effects of empagliflozin on RV diastolic function, though a large variation in end-diastolic elastance (Eed) was seen in the PTB-empa rats.

In patients with established heart failure, no differences were found in the major efficacy outcomes between the different SGLT2 inhibitors, though the different SGLT2 inhibitors have different pharmacodynamic mechanisms (10, 38). The effects of dapagliflozin, another SGLT2 inhibitor, have been investigated in both MCT and PTB models and its function on the RV have proven ambiguous. On one hand Li et al. (26) did not find any protective effects in either MCT or PTB model on the pulmonary vascular resistance or RV function. Connelly et al. (25), too did not find improvements on RVSP, but they suggest osmotic diuresis causing reduced RV weight, as they only found changes in water content of the RV and not reduced RV CSA. In present study on the other hand, there were no changes in RV/(LV + S) weight ratio and only a slight reduction in RV CSA.

### 4.4. RA effects of empagliflozin treatment

RA function did not improve significantly by empagliflozin treatment, but trended according to RA volumes, RA CSA, and RA weight. This too could be a direct consequence of the reduced RV preload and lower RVESP. Others have previously reported reduced RA fibrosis, inflammatory cell aggregation, and ion channeling remodeling in dapagliflozin treated MCT rats with the potential to reduce atrial fibrillation (24).

### 4.5. Empagliflozin treatment in LV vs. RV

Because of the fundamental differences in RV and LV function, it is not possible to directly transfer information gained from the LV to the RV. Empagliflozin seems to be yet another example of this hampered direct translation. Empagliflozin has shown very promising effects in patient with LV heart failure by reducing all-cause mortality (5) and decreasing LV index volumes in patients with type 2 diabetes mellitus (T2DM) and HF with reduced EF (HFrEF) (39). In patients with T2DM and coronary artery disease empagliflozin treatment decreased LV mass index but did not show any impact on RV mass index or RV volumes (40, 41). In an experimental model of HFrEF, treatment with empagliflozin improved LV load-independent contractility and a trend to reduced LV end-diastolic pressure, which again shows the discrepancy between LV and RV response to empagliflozin treatment when comparing with present study (42).

### 4.6. Strengths and limitations

This study has several strengths: (1) PTB produced RV failure similar to that seen in PAH patients by using a well-established pulmonary trunk banding model of isolated RV failure (29). (2) We used several clinically relevant methods to assess RV hemodynamic profile including echocardiography, cardiac MRI, and pressure-volume measurements.

Some limitations must be acknowledged: (1) Only young, male rats without comorbidities were used which differ from the typical patients with PAH. (2) All hemodynamic measures were performed under anesthesia which may blunt the hemodynamic results. This effect was tried minimized by using a well-tested anesthesia protocol. (3) The purpose of present study was to investigate the novel preventive and therapeutic effects of empagliflozin. It was not designed to establish cause and effect of possible mechanisms responsible for the cardiac effects seen by empagliflozin treatment. Further studies are needed for in-dept analyses of the underlying mechanisms. We chose not to directly compare the impact of empagliflozin to commonly used diuretics as it is difficult to match the two diuretics’ effects and as the function of SGLT2 inhibitors differ from traditional diuretics (43). We cannot exclude that some effects might be unconnected to empagliflozin's diuretic effect (25). 4) The rats received empagliflozin mixed in the chow. While it is a less stressful method than for example oral gavage, the received dosage depends on chow consumed. As each cage contained two rats, water and chow consumption for the individual rats were calculated by weight adjusting the consumption measured from the cages. Still, the rats ate more than expected and the empagliflozin dosage were calculated to an average of 30 mg/kg/day. This is a supra-therapeutic dosage of empagliflozin, but in other studies it has been used to investigate its effects and mechanisms in experimental HF (14, 18).

## 5. Conclusion

PTB caused RV failure in all rats subjected to the procedure. Empagliflozin treatment reduced RV end-systolic pressure while maintaining ventricular-arterial coupling. Our study supports that treatment with empagliflozin has no toxic or adverse effects on the failing RV. Further studies are needed to elucidate whether this simply relates to a diuretic effect and/or additional independent beneficial effects on RV function. This will help clarify empagliflozin's potential role in treatment of RV failure.

## Data Availability

The raw data supporting the conclusions of this article will be made available by the authors, without undue reservation.
