# Supplementary material for: Effects of empagliflozin on right ventricular adaptation to pressure overload
Source: Front Cardiovasc Med. 2023 Dec 14;10:1302265. doi: 10.3389/fcvm.2023.1302265 (PMC10757621; doi:10.3389/fcvm.2023.1302265)
Supplement: Supplementary file 1 [file Datasheet1.docx]

Supplementary Material

# Supplementary Figure


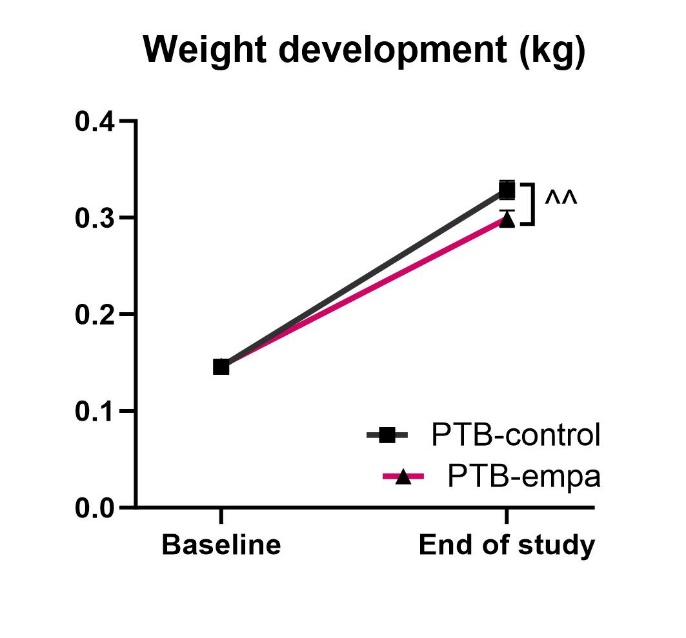


**Supplementary Figure S1: The time effects of empagliflozin treatment on weight development.** PTB: pulmonary trunk banding; empa: empagliflozin. Comparison of rat weight development from baseline to end-of-study in PTB-control and PTB-empa. Results are expressed as 2-way ANOVA. ^^ p < 0.01 PTB-empa vs. PTB-control.


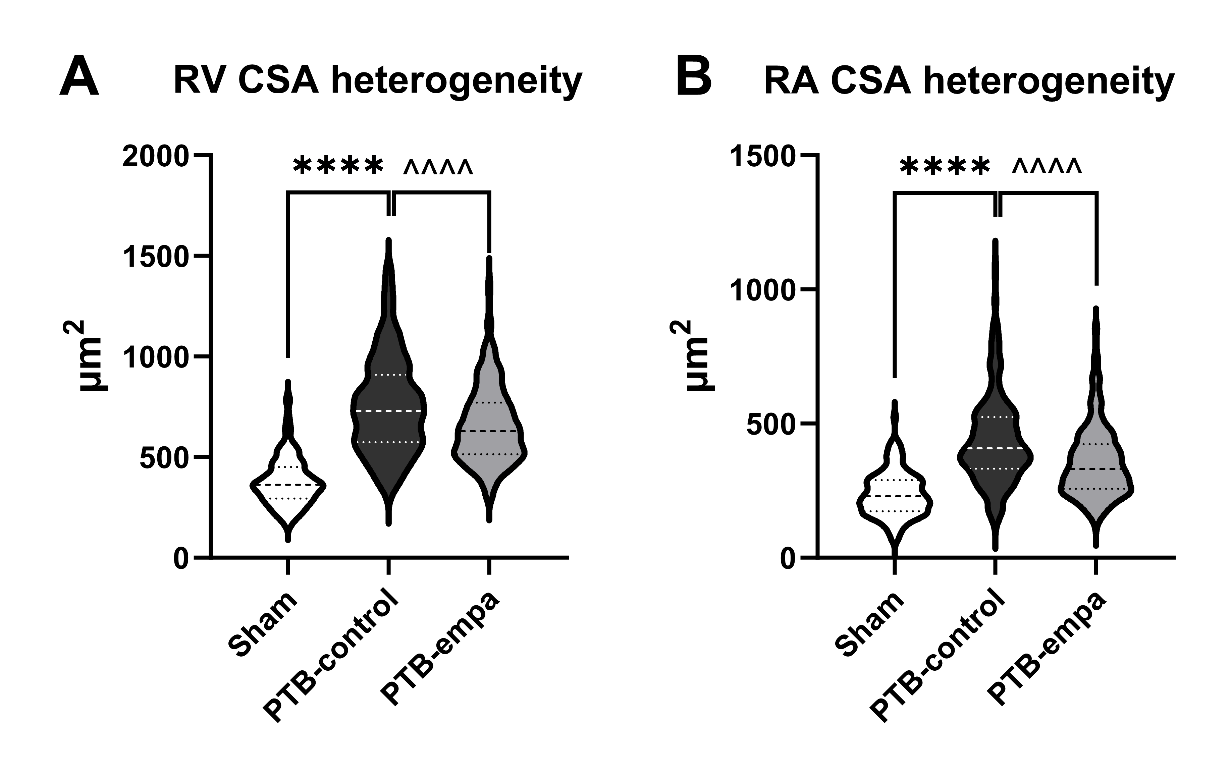


**Supplementary Figure S2: Cardiomyocyte cross sectional area heterogeneity in right ventricle and right atrium.** PTB: pulmonary trunk banding; empa: empagliglozin. A) Right ventricular (RV) cross sectional area (CSA) heterogeneity by pooling all CSA values (sham n=210; PTB-control n=350; PTB-empa n=350). B) Right atrial (RA) CSA heterogeneity by pooling all CSA values (sham n=137; PTB-control n=293; PTB-empa n=295). Results are expressed as violin plots with median and quartiles. **** p < 0.0001 PTB-control vs. sham; ^^^^ p < 0.0001 PTB-control vs. PTB-empa.

# Supplementary Tables

**Table S1: Echocardiography data from one week after pulmonary trunk banding surgery.**

|  | **Sham**  n = 6 | **PTB-control**  n = 10 | **PTB-empa**  n = 10 |
| --- | --- | --- | --- |
| BW week 1 (g) | 146 [145-150] | 141 [138-155] | 146 [134-159] |
| HR (bmp) | 351 [342-382] | 304 [297-328]** | 312 [307-333] |
| CO (mL/min) | 91 [90-121] | 53 [50-56]* | 42 [39-48] |
| Pulm dia (mm) | 2.5 [2.4-2.6] | 2.5 [2.5-2.6] | 2.6 [2.5-2.7] |
| TAPSE (mm) | 2.5 [2.2-2.6] | 1.7 [1.6-1.8]**** | 1.6 [1.5-1.7] |
| RA (mm^2^) | 18 [18-19] | 23 [21-29] | 24 [20-30] |
| E/e’ | 7.0 [5.4-14.8] | 11.0 [10.3-13.4] | 10.4 [9.2-12.1] |
| TR | 0 (0) | 5 (50) | 6 (60) |

PTB: pulmonary trunk banding; empa: empagliflozin; BW: body weight; HR: heart rate; CO: cardiac output; Pulm dia: pulmonary diameter; TAPSE: Tricuspid annular plane systolic excursion; RA: right atrium; E/e’: tricuspid E/e’ ratio; TR: tricuspid regurgitation. Missing data in E/e’ from one PTB-empa due to EA fusion. Results are presented as median [interquartile range] or *n* (%). * p < 0.05; ** p < 0.01; **** p < 0.0001 PTB-control vs sham.

**Table S2: Data at end-of-study.**

|  | **Sham**  n = 6 | **PTB-control**  n = 10 | **PTB-empa**  n = 10 |
| --- | --- | --- | --- |
| Liver (g) | 12.7 [12.4-14.2] | 13.1 [10.7-13.9] | 12.0 [10.5-14.8] |
| Lungs (g) | 1.13 [1.03-1.31] | 1.18 [1.13-1.25] | 1.25 [1.03-1.34] |
| Kidneys (g) | 2.27 [2.10-2.47] | 2.16 [2.00-2.36] | 2.08 [2.01-2.44] |
| Spleen (g) | 0.80 [0.74-0.86] | 0.92 [0.72-1.00] | 0.84 [0.64-0.96] |
| Pulm dia (mm) | 3.1 [3.0-3.4] | 2.8 [2.7-3.0]* | 2.8 [2.7-2.9] |
| Blood potassium (mmol/L) | 5.3 [4.7-5.4] | 5.6 [5.1-6.0] | 5.2 [4.9-5.3] |
| Chloride (mmol/L) | 102.5 [101.5-105.0] | 105.0 [102.0-105.0] | 104.5 [104.0-105.3] |
| Lactate (mmol/L) | 1.20 [0.83-1.65] | 1.20 [1.10-1.35] | 1.35 [1.15-1.58] |
| Bicarbonate (mmol/L) | 31.0 [29.8-32.5] | 29.6 [28.4-30.1] | 29.5 [29.0-30.1] |

PTB: pulmonary trunk banding; empa: empagliflozin; Pulm dia: pulmonary diameter. Results are presented as median [interquartile range]. * p < 0.05 PTB-control vs sham.
